# Treating patients with opioid overdose at a primary care emergency outpatient clinic: a cost-minimization analysis

**DOI:** 10.1186/s12962-021-00303-6

**Published:** 2021-08-04

**Authors:** Jon Hjellum Vibeto, Odd Martin Vallersnes, Andrea Dobloug, Mette Brekke, Dag Jacobsen, Øivind Ekeberg, Knut Reidar Wangen

**Affiliations:** 1 Faculty of Medicine, University of Oslo, Oslo, Norway; 2 Department of General Practice, University of Oslo, Oslo, Norway; 3 Oslo Accident and Emergency Outpatient Clinic, City of Oslo Health Agency, Oslo, Norway; 4 Department of Medicine, Drammen Hospital, Vestre Viken Trust, Drammen, Norway; 5 General Practice Research Unit, University of Oslo, Oslo, Norway; 6 Department of Acute Medicine, Oslo University Hospital, Oslo, Norway; 7 Psychosomatic and Consultation-Liaison Psychiatry, Division of Mental Health and Addiction, Oslo University Hospital, Oslo, Norway; 8 Department of Health Management and Health Economics, University of Oslo, Oslo, Norway

**Keywords:** Poisoning, Opioids, Cost, Primary care, Substance abuse

## Abstract

**Background:**

Treating patients with acute poisoning by substances of abuse in a primary care emergency clinic has previously been shown to be a safe strategy. We conducted an economic evaluation of this strategy compared to hospital treatment, which is the usual strategy.

**Methods:**

Assuming equal health outcomes, we conducted a cost-minimization analysis. We constructed a representative opioid overdose patient based on a cohort of 359 patients treated for opioid overdose at the Oslo Accident and Emergency Outpatient Clinic (OAEOC) from 1.10.2011 to 30.9.2012. Using a health care system perspective, we estimated the expected resources used on the representative patient in primary care based on data from the observed OAEOC cohort and on information from key informants at the OAEOC. A likely course of treatment of the same patient in a hospital setting was established from information from key informants on provider procedures at Drammen Hospital, as were estimates of hospital use of resources. We calculated expected costs for both settings. Given that the treatments usually last for less than one day, we used undiscounted cost values.

**Results:**

The estimated per patient cost in primary care was 121 EUR (2018 EUR 1.00 = NOK 9.5962), comprising 97 EUR on personnel costs and 24 EUR on treatment costs. In the hospital setting, the corresponding cost was 612 EUR, comprising 186 EUR on personnel costs, 183 EUR on treatment costs, and 243 EUR associated with intensive care unit admission. The point estimate of the cost difference per patient was 491 EUR, with a low-difference scenario estimated at 264 EUR and a high-difference scenario at 771 EUR.

**Conclusions:**

Compared to hospital treatment, treating patients with opioid overdose in a primary care setting costs substantially less. Our findings are probably generalizable to poisoning with other substances of abuse. Implementing elements of the OAEOC procedure in primary care emergency clinics and in hospital emergency departments could improve the use of health care resources.

**Supplementary Information:**

The online version contains supplementary material available at 10.1186/s12962-021-00303-6.

## Background

Acute poisoning by substances of abuse is a major health problem [1, 2], entailing substantial costs [3–7]. From 2000 to 2014 the rate of deaths from drug overdoses rose by 137% in the United States, including a 200% increase in the rate of deaths related to opioid overdoses [8]. The total economic burden of opioid overdose in the United States was estimated at 20 billion USD in 2009, including 2.2 billion USD in direct medical costs [9].

Rising health care costs is a major political issue in all developed countries [10]. Since prioritizing is necessary, it is important that the resources used are applied in a cost-effective way. A main cost-containing principle in the health care sector is to treat the patients at the lowest effective level of care [11].

The level of hospital treatment has a major impact on the cost. In a Belgian study, treating acute poisoning in an intensive care unit (ICU) cost three times as much as in an emergency department observation unit [12]. In Oslo, Norway, a large number of patients with acute poisoning by substances of abuse, one out of four involving opioids, are not treated in a hospital at all, but in a primary care setting, at the Oslo Accident and Emergency Outpatient Clinic (OAEOC) [13–15]. Treatment at the OAEOC is based on a local procedure, previously shown to be safe [16]. In most other European cities, patients with acute poisoning are mainly treated in emergency rooms in hospitals that have more advanced diagnostic and treatment possibilities than those available in the primary care setting at the OAEOC.

Cost analyses of different treatment regimens for acute poisoning are scarce. A US study found only a small reduction in the economic burden on hospital emergency departments by triaging patients with uncomplicated ethanol poisoning to designated sobering centres, as 80% of the patients also needed inpatient services not available at the sobering centre [17]. However, among patients treated for acute poisoning by substances of abuse at the OAEOC, no more than 17% were transferred to hospital [16]. Hence, the OAEOC procedure would seem an alternative to hospital treatment. The direct medical costs associated with treating patients with acute poisoning at the OAEOC have not previously been calculated. To complement our previous work showing the safety of the OAEOC procedure [16, 18, 19], we now set out to perform an economic evaluation of this treatment strategy.

### Aims

From a health care system perspective, we compared the costs of treating a patient with opioid overdose in the primary care setting at the OAEOC with the costs of treating a patient with the same characteristics in a hospital, exemplified by Drammen Hospital, a large Norwegian general hospital.

## Methods

We conducted a cost-minimization analysis to compare the expected costs of treating a constructed representative patient with opioid overdose at the two sites. A cost-minimization analysis is an economic evaluation resting on the assumption that the outcomes of the two treatment regimens are equal despite the different diagnostic and therapeutic possibilities in the two settings [16, 20].

### Settings

The emergency care system in Norway has two levels; primary care emergency services and hospitals (specialist care). Patients usually cannot present directly to hospitals; they first have to be assessed by the ambulance service or in primary care. Primary care emergency services are provided by regular general practitioners during office hours and at local primary care emergency clinics at other times.

The OAEOC is the main primary care emergency clinic in Oslo, encompassing a trauma clinic and a general practice service. Most doctors are registrar general practitioners. There are about 200,000 consultations per year. The population of Oslo was 673,000 in 2018 [21]. Oslo is the capital city of Norway, and the population within the municipality is urban. The OAEOC differs from primary care emergency clinics in other Norwegian cities, most notably in that it is open at all hours, has the capacity to observe patients for up to 24 h, and has computed tomography (CT) head scan available. With these exceptions, the general practice service at the OAEOC is a standard primary care facility with limited diagnostic resources.

Drammen Hospital is a general hospital and the largest entity in the Vestre Viken Hospital Trust. It serves as a local hospital for a population of 168,000 and has several regional functions for the Vestre Viken region population of almost 500,000 [22]. About two thirds of the local hospital population can be considered urban.

### Opioid overdose

Patients with opioid overdose typically present with a triad of reduced level of consciousness, respiratory depression, and miotic pupils [23, 24]. Treatment focuses on achieving adequate respiration, with respiratory support and/or the antidote naloxone [23, 24]. The effect of naloxone is shorter than for most opioids, including heroin, hence the patient should be observed for 2 h after naloxone administration, to treat any recurring respiratory depression [25]. About 500–800 opioid overdoses are treated at the OAEOC per year [15, 26], and an estimated 20 at Drammen Hospital.

### Procedure at the OAEOC

The procedure in use at the OAEOC for managing patients with poisoning by substances of abuse has been developed locally [16]. At arrival, the patient is triaged by a nurse (using the Manchester Triage System [27]), who initiates the procedure if poisoning by substances of abuse is suspected. A nurse and a registrar doctor are assigned to the patient, and the nurse will perform initial measurements, including respiratory rate, SpO_2_, heart rate, blood pressure, temperature, blood glucose, and Glasgow Coma Scale (GCS) score, as well as noting the case history. The doctor then examines the patient by at least auscultating the heart and lungs, checking for track marks, signs of injury, pupil size and light reaction, spontaneous nystagmus, and plantar reflex. Point of care measurement of haemoglobin and C-reactive protein (CRP) are available, as are electrocardiogram (ECG), conventional X-ray, and CT head scan. Blood gas analysis is not available, and generally no laboratory analyses are performed.

**Fig. 1:**
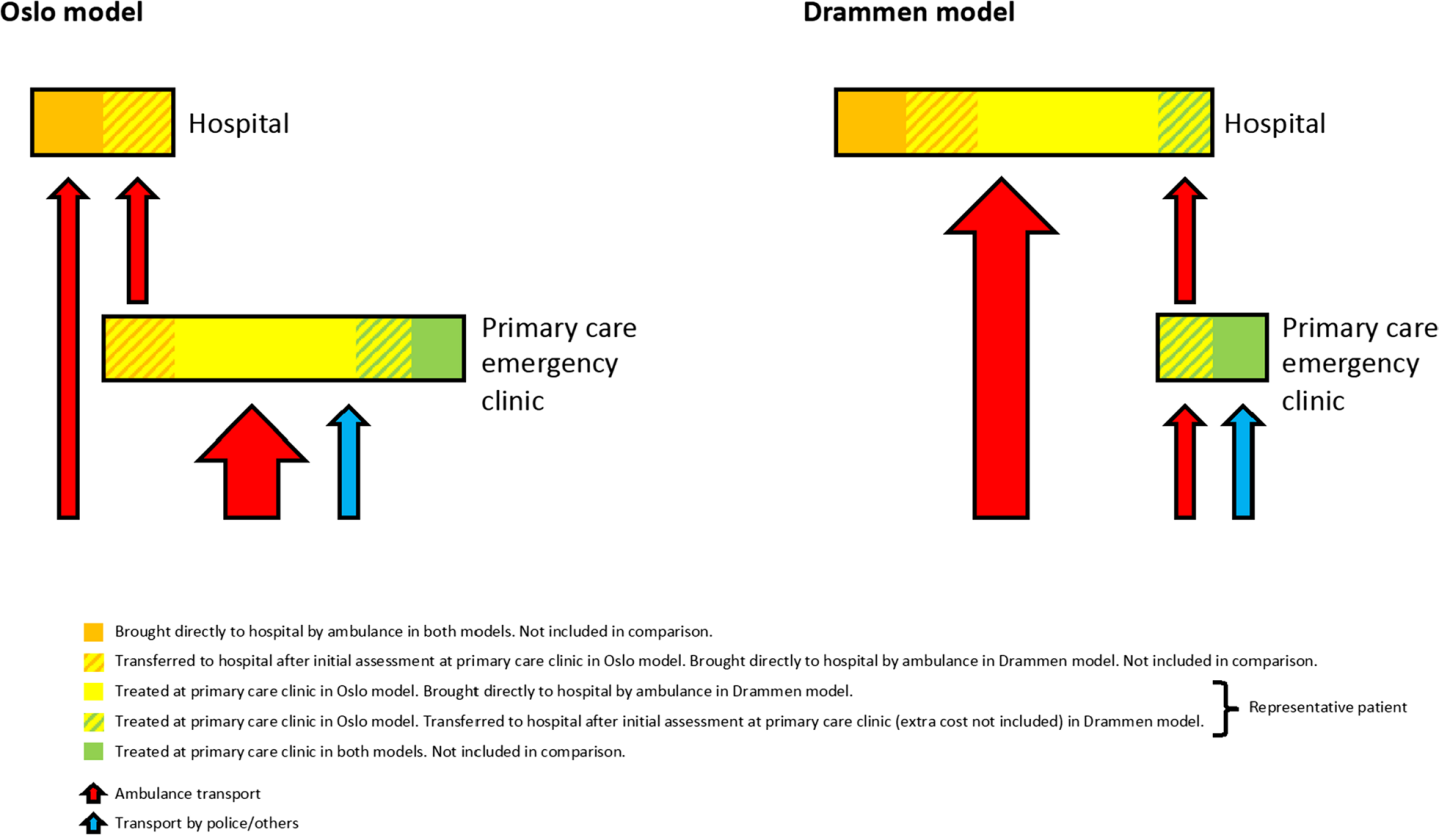
Patient flow for treating patients with opioid overdose in primary care or hospital. Patient flow for treating patients with opioid overdose mainly in a primary care emergency clinic (Oslo model) or at a hospital (Drammen model)

After the initial examination, the doctor decides whether the patient should be transferred to hospital, observed at the OAEOC, or discharged (Fig. 1). In case of respiratory depression (local definition: respiratory rate < 10/min and SpO_2_ < 90%), the doctor should administer naloxone if opioid overdose is suspected and keep the patient for observation for at least 2 h. Patients with respiratory depression not responding to antidote are transferred to hospital (flumazenil is also available, but rarely used, and patients needing flumazenil will be transferred to hospital for further treatment), as are patients with GCS < 7, hyperthermia, psychosis, or other conditions needing more diagnostic or treatment resources than available at the OAEOC. Patients kept at the OAEOC are observed by the assigned nurse every 15–30 min, noting GCS score, pupil light reaction, respiratory rate, SpO_2_, and symmetric movement of arms and legs. When necessary, the nurse consults the doctor. Patients who are deteriorating or not alert within 4 h are transferred to hospital, the rest are discharged by the doctor. A senior registrar doctor is always available if needed.

### Clinical course at Drammen Hospital

A likely course of treatment for a patient with suspected opioid overdose at Drammen Hospital would start with the patient being brought to an emergency room, especially in case of reduced consciousness or respiratory depression. The patient is handled by two nurses and two doctors (an intern and a senior registrar). Oxygen therapy is often initiated immediately, and intravenous access established. Fluid therapy (Ringer acetate) is frequently initiated. If opioid overdose is suspected, many patients are treated with naloxone. Flumazenil is also often used. Almost all patients have an ECG taken and blood samples drawn for a standard set of blood tests and arterial blood gas analysis. A CT head scan is often done if the diagnosis is uncertain after the initial assessment. Assuming nothing substantial has been unveiled so far, the next step mainly depends on the respiratory status of the patient. If the patient still has respiratory depression, or is considered unstable, the patient is admitted to an ICU. The alternative is admission to a general ward, with less monitoring possibilities. During daytime and evening, the ICU has one nurse for every patient, at night there are four nurses in charge of a maximum of six patients. Treatment at the ICU ranges from mere observation to full-scale intensive treatment, depending on the severity of the clinical condition. Many patients admitted to the ICU in Drammen due to opioid overdose do not need any more specific medical treatment but are in need of observation, and hence admitted to enable continuous surveillance of their respiration. The patients are typically directly discharged from the ICU. In general, they are allowed to stay until they wake up and leave on their own.

### Constructing a representative patient

From an original cohort of 2343 cases treated for acute poisoning by substances of abuse at the OAEOC from 1 October 2011 to 30 September 2012, we extracted the 539 cases with clinically suspected opioid overdose. Among them, 102 were transferred to hospital, and therefore not included in this analysis (Fig. 1). Furthermore, 78 never had a GCS score below 15, and we assumed that these patients would have been treated at the primary care emergency clinic in Drammen, and not at the hospital (Fig. 1). This yielded 359 cases of opioid overdose, treatable in primary care using the OAEOC procedure and treated in hospital in Drammen.

We had data available on age, gender, mode of presentation, toxic agents taken, clinical observations, complications, treatment, length of stay, and disposition.

Based on the available information for the 359 included cases, we constructed a representative patient: *He is a 38 years old male brought by ambulance with a suspected opioid overdose. He has a respiratory depression with a respiratory rate* < *10/min, but SpO*_*2*_ > *90%. He is brought in the morning at the beginning of the dayshift and observed at the OAEOC for 4 h 45 min before being roused and discharged. The lowest GCS recorded during observation is 12.*

The constructed representative patient was deemed to have respiratory depression though this was only seen in 36% of the cases while at the OAEOC. However, in total 28% were treated with naloxone, most of them by the ambulance before arrival at OAEOC. Hence, we assumed that most patients had respiratory depression at some point.

### Estimating resources used—OAEOC

The expected resources used on the representative patient encompass personnel resources (the amount of time used by staff) and test/treatment resources (Table 1). The time used by the staff at the OAEOC was estimated jointly by JHV, OMV and local informant MN. The probabilities of using the test/treatment resources were estimated jointly by JHV and OMV based partly on specific data for the 359 opioid overdose cases and partly on data for the whole OAEOC cohort of 2343 cases treated for poisoning by substances of abuse. A decision tree illustrating the decisions made by the doctor at the OAEOC is included in Additional file 2: Figure S1.

**Table. 1:** Estimated resources used and unit costs for treating a patient with opioid overdose

Oslo Accident and Emergency Outpatient Clinic			Drammen Hospital		
**Personnel**	**Cost per hour EUR**	**Minutes spent**	**Personnel**	**Cost per hour EUR**	**Minutes spent**
Triage nurse	34 (31–37)	6 (3–12)	Nurse 1	32 (30–34)	90 (75–105)
Observation nurse	34 (31–37)	90 (75–105)	Nurse 2	32 (30–34)	90 (75–105)
Registrar	49 (45–53)	45 (30–60)	Intern	40 (37–43)	60 (45–75)
Senior registrar	56 (53–58)	6 (3–12)	Senior registrar	50 (47–52)	60 (45–75)
**Test/treatment**	**Unit cost EUR**	**Probability of use**	**Test/treatment**	**Unit cost EUR**	**Probability of use**
Glucose	16	0.98 (0.96–1.00)	Blood tests^a^	67	0.99 (0.98–1.00)
CRP	9	0.10 (0.05–0.20)	Arterial blood gas	30	0.90 (0.80–1.00)
ECG	44	0.03 (0.01–0.05)	ECG	44	0.90 (0.85–0.95)
CT head scan	86	0.06 (0.04–0.08)	CT head scan	86	0.40 (0.30–0.55)
Naloxone	6	0.10 (0.05–0.15)	Naloxone	6	0.80 (0.70–0.90)
			Flumazenil	15	0.50 (0.40–0.60)
			Ringer acetate	5	0.50 (0.40–0.60)
			**Intensive care unit**		
			**Price per 24 h EUR**	**Hours of stay**	**Probability of use**
			1042 (834–1250)	8 (6–10)	0.70 (0.60–0.80)

Brackets display range of uncertainty

CRP: C-reactive protein; CT: computed tomography; ECG: electrocardiogram, EUR: European euro

^a^Battery of blood tests shown in Additional file 1: Table S1

### Estimating resources used—Drammen Hospital

Time used on the representative patient by the staff at Drammen Hospital was estimated jointly by AD and local informants BHH and TB (Table 1). The estimated times do not include time used on the patient in the ICU. The probabilities of using the test/treatment resources were estimated jointly by JHV and AD. The resulting model was presented to a group of other doctors and nurses at the Emergency Department at Drammen Hospital who made their own predictions of treatment pathway and probabilities. These estimates were incorporated in the final model. Our patient would most likely be treated with naloxone, with a combined diagnostic and therapeutic intention. Limited effect of naloxone and/or uncertain diagnosis could lead to flumazenil being administered and/or a CT head scan being done. We estimated 90 min for the initial examinations, tests, and subsequent CT head scan. At this point the patient would probably still be somnolent, with or without respiratory depression, and in need of further observation in the ICU. We assumed the patient to stay in the ICU for 8 h, without receiving any specific treatment, and then leaving the hospital without further investigations. A decision tree illustrating the clinical pathway at Drammen Hospital is included in Additional file 3: Figure S2.

### Estimating costs—general

We used the Norwegian Directorate of Health’s guide for economic evaluations in the health care sector, based on the principle that resources should be valued according to their best alternative use, the opportunity cost [28]. Accordingly:

- Personnel costs were estimated based on the average pay of the personnel involved, multiplied by 1.3 to count in payroll taxes and other social costs [29].
- For services provided by hospitals, unit costs were estimated as if financed by full reimbursements from The Norwegian Health Economics Administration (Helfo).
- For services provided by general practitioners and other specialists, unit costs were estimated by multiplying the reimbursements from Helfo [16, 30] by two, to count in other forms of financing, e.g., funding from the Municipality of Oslo. This approximation to the actual unit costs is according to the national recommendations [28].
- For outpatient radiological and laboratory services, unit costs were estimated as the reimbursed sum from Helfo plus the fee payed by patient, multiplied by two [28].

Costs were estimated using 2018 figures (2018 EUR 1.00 = NOK 9.5962). We used undiscounted costs because both the alternative treatments usually last for less than one day.

### Estimating costs—OAEOC

Average hourly wages at the OAEOC were estimated jointly by JHV and local informants JØ and PCH (Table 1). Tests were defined as outpatient radiological and laboratory services and the unit costs estimated accordingly [30–32]. Unit cost for naloxone was set to the retail price [33].

### Estimating costs—Drammen Hospital

Average wages for the nurses at the Emergency Department at Drammen Hospital were estimated jointly by AD and local informant TB. Average seniority and wages for the doctors were estimated by AD based on the agreement between Vestre Viken Hospital Trust and The Norwegian Medical Association [34].

The Norwegian reimbursement system defines a patient staying in hospital for less than 5 h as an outpatient [35]. Our representative patient stays for longer than that. Cost estimates could have been obtained using the fixed sums used for admitted patients. However, those sums are mostly based on registered diagnoses and invasive procedures and using them would make it difficult to isolate the direct medical costs in Drammen and entail diverging methods of calculating costs at the two facilities. Thus, to make the calculations more comparable, we chose to use Helfo outpatient reimbursements for each service provided.

The sums reimbursed by Helfo for blood tests (Additional file 1: Table S1), blood gas analysis, and CT head scan were obtained from the Department of Laboratory Medicine at Drammen Hospital [31, 32, 36]. We did not succeed in finding the reimbursement for a hospital ECG and chose to use the same sum as at the OAEOC [30]. Unit costs for naloxone, flumazenil and Ringer acetate were set to the retail prices [33]. The personnel costs of a bioengineer for the blood samples and radiograph and radiologist for the CT head scan are included in the unit costs for the blood samples and CT head scan, respectively.

Searching the literature, we did not find estimates for the cost of treating uncomplicated patients in an ICU. JHV, AD, and local informant BHH, head nurse at the ICU in Drammen, jointly settled on an estimate of 1 042 EUR per 24 h for our representative patient. The estimate included a broader set of costs (both direct and indirect) than the direct costs estimated at OAEOC and the Emergency Department in Drammen. The senior doctor would most likely use time treating the patient in the ICU. This time was not included in the personnel costs, but in the total cost of intensive care treatment.

### Calculation of expected costs

Expected personnel costs were calculated by multiplying personnel costs per hour with expected time spent. Expected treatment costs were calculated by multiplying the unit cost with the probability of the test/treatment being done. Expected costs of intensive care treatment were calculated by multiplying the probability of ICU admission with the estimates for price and length of stay.

### Sensitivity analyses

Reimbursements from Helfo and prices on medicines are considered actual. All other parameters are stated with attached uncertainties estimated based on information from key informants on provider procedures at both institutions. Sensitivity analyses were carried out to assess the possible impact of the uncertainties on the results. We conducted one-way analyses where parameters were varied one at a time. The sensitivity analyses are presented in tornado diagrams as described by Drummond et al. [20].

## Results

**Fig. 2:**
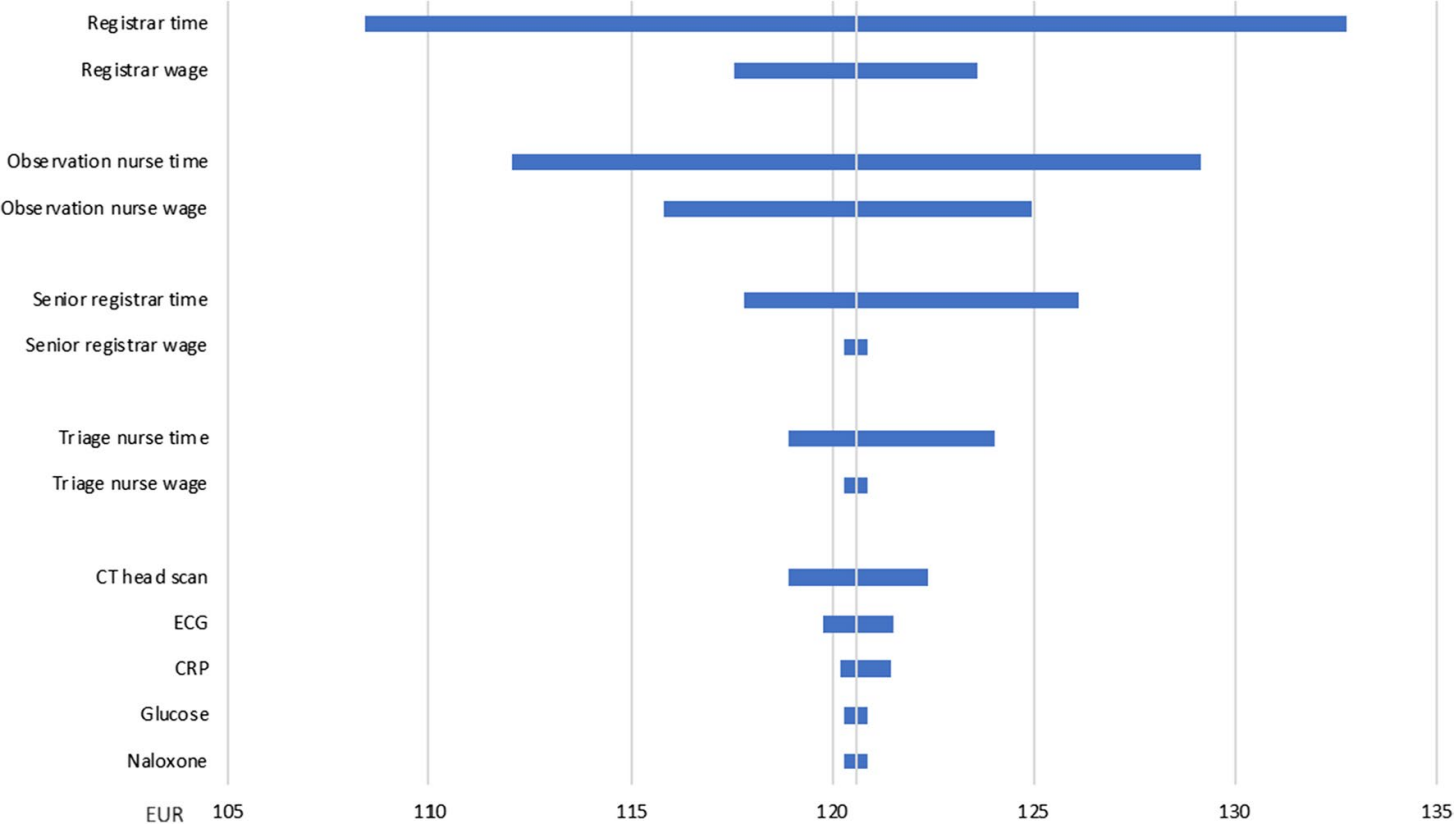
Sensitivity analysis for primary care outpatient clinic treatment. One-way sensitivity analysis of expected costs for treating a patient with opioid overdose at the Oslo Accident and Emergency Outpatient Clinic, showing highest and lowest values of the parameter in question. CRP: C-reactive protein; CT: computed tomography; ECG: electrocardiogram; EUR: European euro

The point estimate of the expected costs of treating a representative patient with opioid overdose at the OAEOC was 121 EUR, split between personnel costs of 97 EUR and test/treatment costs of 24 EUR (Table 2). In the sensitivity analysis, the highest estimate for the total costs was 165 EUR, and the lowest estimate was 88 EUR. The uncertainties concerning resources used by the assigned nurse and registrar doctor were most important, with time spent being more important than wages (Fig. 2).

**Table. 2:** Expected costs for treating a patient with opioid overdose

Oslo Accident and Emergency Outpatient Clinic				Drammen Hospital			
	Expected costEUR	Low valueEUR	High valueEUR		Expected costEUR	Low valueEUR	High valueEUR
**Personnel**				**Personnel**			
Triage nurse	3	2	7	Nurse 1	48	38	60
Observation nurse	51	39	65	Nurse 2	48	38	60
Registrar	37	23	53	Intern	40	28	54
Senior registrar	6	3	12	Senior registrar	50	35	65
Sum	97	67	137	Sum	186	139	239
**Test/treatment**				**Test/treatment**			
Glucose	16	15	16	Blood tests^a^	66	66	67
CRP	1	1	2	Arterial blood gas	27	24	30
ECG	1	1	2	ECG	40	37	42
CT head scan	5	3	7	CT head scan	34	26	47
Naloxone	1	1	1	Naloxone	5	4	5
Sum	24	21	28	Flumazenil	8	6	9
				Ringer acetate	3	2	3
				Sum	183	165	203
				**Intensive care unit**	243	125	417
**Total costs**	121	88	165	**Total costs**	612	429	859

Low/high value: Lowest/highest achievable value given the assumptions

CRP: C-reactive protein; CT: computed tomography; ECG: electrocardiogram, EUR: European euro

^a^Battery of blood tests shown in Additional file 1: Table S1

**Fig. 3:**
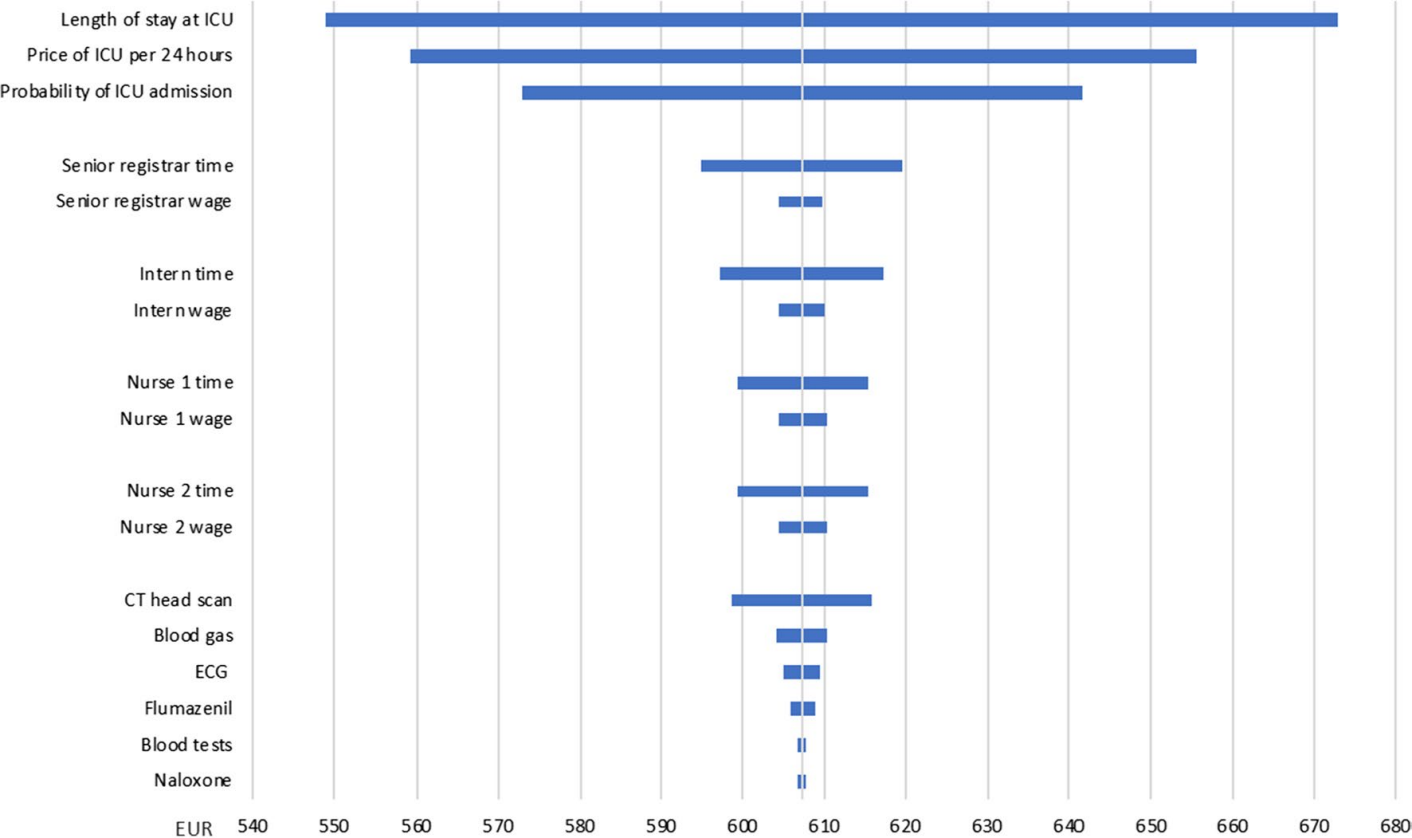
Sensitivity analysis for hospital treatment. One-way sensitivity analysis of expected costs for treating a patient with opioid overdose at Drammen Hospital, showing highest and lowest values of the parameter in question. CT: computed tomography; ECG: electrocardiogram; ICU: intensive care unit; EUR: European euro

The point estimate of the expected costs of treating the same patient at Drammen Hospital was 612 EUR. The personnel costs were 186 EUR, test/treatment costs 183 EUR and the cost of intensive care 243 EUR (Table 2). In the sensitivity analysis, the highest estimate for the total costs was 859 EUR, and the lowest estimate was 429 EUR. The uncertainties concerning costs associated with the ICU were the most important factors, followed by the time used by staff, and the probability of conducting a CT head scan (Fig. 3).

**Fig. 4:**
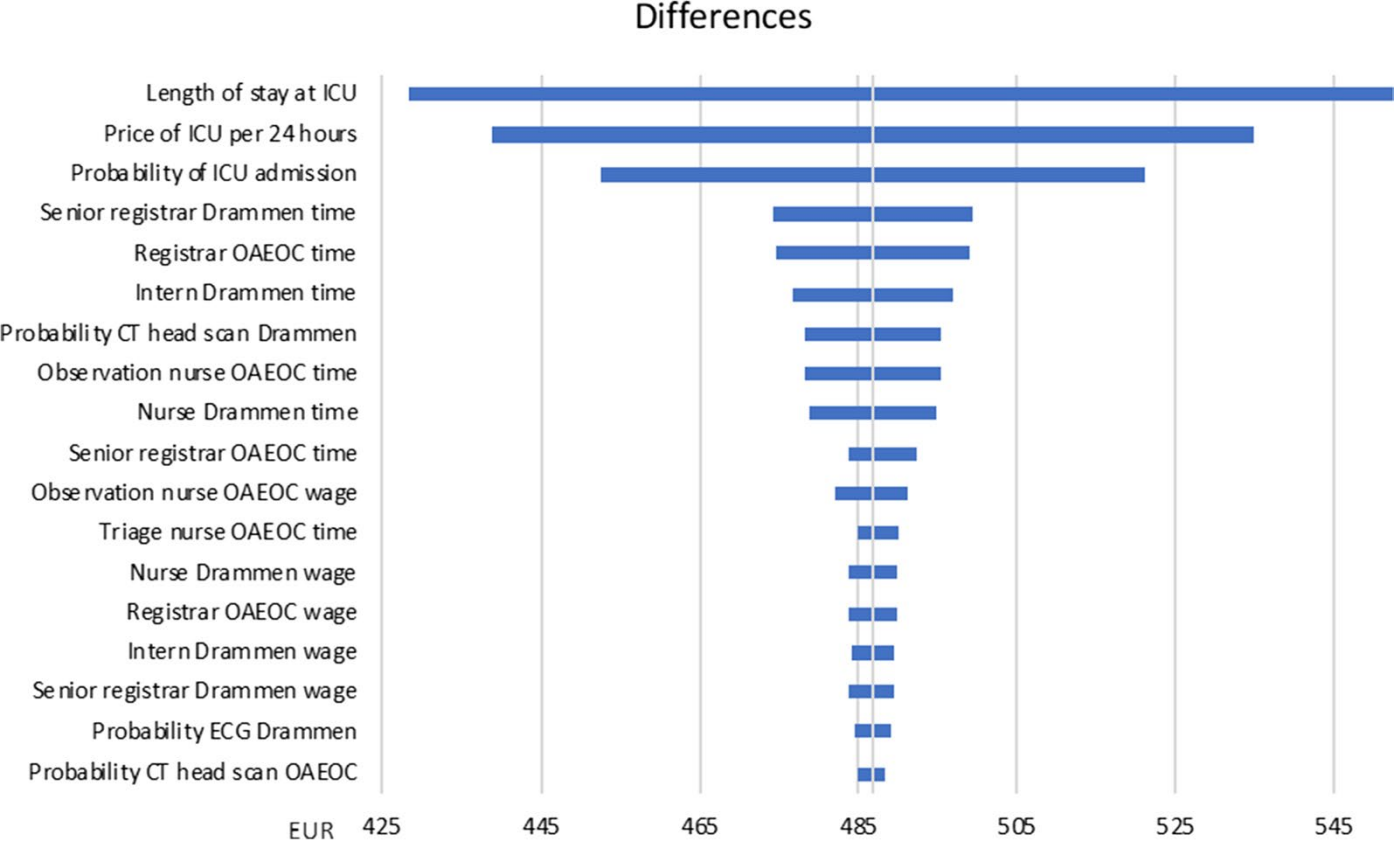
Sensitivity analysis for cost difference between primary care outpatient clinic and hospital treatment. One-way sensitivity analysis of the different parameters on the difference in expected costs for treating a patient with opioid overdose at the Oslo Accident and Emergency Outpatient Clinic (OAEOC) and at Drammen Hospital. Parameters with minor importance are left out. CT: computed tomography; EUR: European euro; ICU: intensive care unit; OAEOC: Oslo Accident and Emergency Outpatient Clinic

The point estimate of the difference between Drammen Hospital and the OAEOC was 491 EUR, split on 243 EUR on ICU costs, 89 EUR on personnel costs and 159 EUR on test/treatment costs (Table 2). The highest possible difference was 771 EUR, while the lowest possible difference was 264 EUR. In the sensitivity analysis the most important parameters were associated with the ICU (Fig. 4).

## Discussion

Treating a representative patient with opioid overdose in primary care at the OAEOC cost substantially less than treating the same patient in a hospital setting at Drammen Hospital. The point estimate of the difference of expected costs was 491 EUR per patient, making hospital treatment five times more expensive. Our representative patient was based on 359 patients treated for opioid poisoning during one year, yielding a total saved cost in Oslo per year of 176,269 EUR.

Sensitivity analyses revealed major uncertainties concerning the probability of admission to the ICU, and the price and length of that stay. However, the uncertainties did not challenge the main results, and the difference was substantial (264 EUR) also in the lowest possible estimate of the difference, with hospital treatment still more than twice as expensive. Furthermore, our estimate for the ICU cost is lower than what was found for poisoned ICU patients in general in studies from Belgium and Spain [6, 12], for opioid overdose ICU patients in a Canadian study [37], and for general ICU patients not requiring any expensive interventions in an Irish study [38]. Our point estimate for the cost of treating patients with opioid overdose at the OAEOC is in the same range as found for emergency department ambulatory care for poisoned patients in general in the Belgian and Spanish studies [6, 12]. Another Belgian study found the cost of treating alcohol intoxicated patients in a hospital emergency department in the same range as our point estimate for hospital treatment of opioid overdoses [39].

A cost-minimization analysis rests on the assumption that the outcomes of the two treatment regimens are equal, implying that the extra resources used per patient in the hospital setting do not produce any additional positive health effect. At the hospital, our representative patient would not receive any treatment directed at the opioid overdose that is not available at the OAEOC. Moreover, in a study of 1952 discharges from the OAEOC, only 13 patients (0.7%) re-presented to the OAEOC or any Norwegian hospital with a diagnosis overlooked at the initial observation; no patients died at the OAEOC, but two patients died of a new opioid overdose the following week [16]. Still, questions may be raised concerning the assumption of equal outcomes in our study. First, 19% of the patients brought to the OAEOC with suspected opioid overdose were transferred to hospital [16]. Some of these patients might have profited by being brought directly to hospital without the intermediate stop at the OAEOC, though none of them died while admitted [16]. Second, patients treated for opioid overdose have a high rate of co-morbidity [40]. Through supplementary tests at the hospital, conditions that otherwise would have gone without recognition for some time, could be diagnosed and possibly treated. However, we find it likely that many of the patients in such need were among the opioid overdose patients transferred from the OAEOC to hospital. In a previous study, four out of ten patients transferred from the OAEOC were discharged from the hospital with another main diagnosis than acute poisoning [16]. Finally, the availability of a liaison psychiatry service might facilitate follow-up after discharge from the hospital. However, referral to specialist psychiatric or addiction health services after treatment for substance use related poisoning is not more frequently arranged at hospitals than at the OAEOC, and in a previous study, 85% of the patients thus referred from the OAEOC attended [18, 41]. In sum, we consider the assumption of equal outcomes tenable in our study.

There is no single aspect explaining the difference in the treatment regimens. The ample diagnostic and treatment possibilities at the hospital enable a culture where they are used. The main concern for the doctor treating a patient with reduced consciousness is to overlook differential diagnoses, and many of the tests performed are done to ensure not making that mistake. At the OAEOC, opioid overdose is a frequent event [15, 26]. Hence, OAEOC staff could be more comfortable in trusting their judgement of this clinical condition than staff at Drammen Hospital, where opioid overdoses are rarer. Then again, the majority of patients brought to the OAEOC with reduced consciousness suffer from acute poisoning, while patients with reduced consciousness brought to the emergency department at Drammen Hospital is a more varied group, necessitating a broader approach. With this caution, adopting the OAEOC procedure in hospital emergency departments could be possible. The procedure has been shown to be safe [16], and all the necessary diagnostic tools and treatments are available at the hospital.

Another option would be to treat these patients at the primary care level. The OAEOC procedure does not require any equipment or type of personnel beyond what is found in any Norwegian primary care emergency clinic. Though smaller such clinics would not have enough manpower nor enough relevant patients, several cities have populations sufficiently large to implement the procedure. If we consider one patient per day with acute poisoning by substances of abuse sufficient to keep staff trained, a catchment area population of 100,000 would allow for the use of the procedure (2343 cases at the OAEOC in one year yields 6.4 per day in a population of 613,000 in Oslo in 2012 [16]). Distance to the nearest hospital would also limit observation possibilities in primary care—the further away, the lower the threshold should be for transfer. Implementing two-tiered emergency care in other countries might also allow for primary care use of the OAEOC procedure.

As one of the aspects enabling an efficient treatment of patients with opioid overdose at the OAEOC is the sheer volume, transferring treatment of this large patient group to Oslo hospitals might have led to the development of leaner procedures than in Drammen. Hence, estimating the amount of money saved by treatment at the OAEOC is not as simple as multiplying our cost difference with the relevant number of cases.

We chose patients with opioid overdose for our analysis, as opioids are the second most frequent toxic agent seen at the OAEOC, after ethanol [13–15]. Comparing costs of treating patients poisoned by other substances of abuse than opioids would probably yield similar results, as the same procedure is used. However, cost differences would be smaller, e.g. for ethanol poisoning. Patients with ethanol poisoning are rarely transferred to hospital from the OAEOC [16]. Respiratory depression is not a common feature among these patients, and few of them would be admitted to an ICU. Still, most of the other treatments would probably be used at the hospital, making hospital treatment cost more for this patient group as well.

### Strengths and limitations

For simplicity, we omitted capital costs and overhead costs from the analysis. Capital costs, such as buildings and land, are encountered at a specific point in time [42]. Overhead costs are costs shared by more than one department or entity, e.g. costs of information and communications technology and caretaker services [42]. Even though these types of costs belong in a full economic evaluation, the group of patients under scrutiny here is sufficiently small (acute poisoning constitute about 1.5% of total cases at the OAEOC) for variable costs to be overwhelmingly more important.

Omission of per diem costs at the OAEOC is somewhat more problematic. The per diem cost is described as the hotel aspect of staying in a health institution, excluding the direct medical costs of drugs and special consumable items [42]. The hotel cost of treatment at the emergency department in Drammen is negligible and safely omitted. It seems safe to consider per diem costs to be small at the OAEOC as well, even though the patient stays for more than 4 h, and sometimes is served a little food. However, per diem costs were included in the estimates from the ICU. Among these three units, the hotel costs are arguably most relevant at the ICU, but including one type of costs in the analysis in Drammen and not at the OAEOC is a possible source of overstating the costs in Drammen, though the omitted costs at the OAEOC is expected to be small.

The uncertainties of the model are explored through one-way sensitivity analyses, presented in tornado diagrams. A limitation of this approach is that one-way sensitivity analyses do not address the combined variability of the different parameters. This limitation was remedied by maximizing all parameters at the OAEOC and minimizing all parameters in Drammen (and vice versa). In this extreme scenario, the expected costs in Drammen still were 2.6 times higher than at the OAEOC, which is a key finding in this study.

A strength of the study was the utilization of real-world data from the OAEOC to construct a representative patient, i.e. a patient treated for opioid overdose at the OAEOC, treatable in primary care, who would have been treated in hospital elsewhere.

We did not include the patients transferred from the OAEOC to hospital in our analyses (Fig. 1), as they were deemed too sick for primary care management. Hence, they were more severely sick than the representative patient and would probably have received more intensive treatment than this patient at Drammen Hospital. Among patients with poisoning involving opioids transferred from the OAEOC to an Oslo hospital, 93% were admitted to the ICU [43]. This is higher than our estimate of a 70% probability (range 60–80%) for ICU admittance for the representative patient at Drammen Hospital. Still, resources were spent at the OAEOC assessing and/or observing them. For the 359 patients successfully treated at the OAEOC in our study, 102 were transferred. If we were to assume that these patients received the same treatment at Drammen Hospital as the representative patient, the OAEOC cost should be increased by 102/359 (28.4%). This would yield a point estimate of 155 EUR and a high estimate of 214 EUR, still half the cost of the lowest hospital estimate (429 EUR). Hence, this simplification does not challenge our main results.

Furthermore, some patients were transferred to Drammen Hospital after initial assessment at the local primary care emergency clinic (Fig. 1). These patients were included in the study, but we did not include the cost of the primary care assessment of these patients. At the primary care emergency clinic in Drammen, this assessment would probably constitute a summary clinical examination and rapid triage for transfer to hospital, using less personnel and test/treatment resources than at the OAEOC. Not adding this cost to the Drammen model may somewhat underestimate the cost difference, strengthening the robustness of our main results.

Transportation costs were not included in our comparisons, as they would be similar in the two models (Fig. 1). All patients were transported once, and we do not have reason to believe that the proportions not transported by ambulance were different in the two settings. The patients needing a second transport for transferral from the OAEOC to hospital were not included in the study, as discussed above. Transportation costs would have increased the adjusted cost at the OAOEC if these patients were to be included. On the other hand, the patients needing a second transport in Drammen were included. We do not know how many these patients were, and hence did not estimate the extra transportation cost. Not including this cost underestimates the Drammen costs, but strengthens our conclusion.

The probabilities of using the different tests and treatments at the OAEOC were based on real world data evaluated by key informants—all experienced local clinicians. The estimations of time spent by the staff are more uncertain as they were solely based on the assessments of experienced local clinicians. Estimates of resources used at Drammen Hospital were entirely based on the assessments of key informants—also all experienced local clinicians. The scenario was worked through numerous times and then adjusted after discussion with a group of other experienced local clinicians. Clinicians’ best guess can never be a perfect substitution for real world data, but the external group of local clinicians were very much in line with our original estimates, making it likely that these estimates are reliable.

Though we constructed our representative patient on data from 2012, the procedure at the OAEOC is unchanged, and cost estimations were done using 2018 figures. Heroin is still by far the most common opioid taken in opioid overdose at the OAEOC, though longer acting opioids have appeared more frequently since 2017 [26].

## Conclusions

Treating patients with opioid poisoning in a primary care setting costs substantially less than treating a patient with similar characteristics in a hospital, with estimated total costs at 121 EUR and 612 EUR, respectively. Even when comparing the highest cost estimate in primary care, 165 EUR, with the lowest cost estimate at the hospital, 429 EUR, the difference of 264 EUR is substantial. Elements of the OAEOC procedure could be implemented in hospitals and primary care emergency clinics both in Norway and abroad, saving health resources.

To corroborate our results, future research could collect real-life data from hospital settings, both concerning time spent by involved personnel and the probabilities of the use of tests, treatments, and intensive care, as well as manually measure personnel resources used in both settings.

## Supplementary Information

**Additional file 1: Table S1.** Blood tests at hospital.

**Additional file 2: Figure S1.** Decision tree at outpatient clinic.

**Additional file 3: Figure S2.** Decision tree at hospital.

## Data Availability

The datasets generated and analysed during the current study cannot be made openly available due to conditions set by the Regional Committee South East for Medical and Health Research Ethics prior to collecting the data. Inquiries about the data and conditions for access can be made to the corresponding author.

## Abbreviations

CRP: C-reactive protein
CT: Computed tomography
ECG: Electrocardiogram
EUR: European euro
GCS: Glasgow Coma scale
Helfo: The Norwegian Health Economics Administration
ICU: Intensive care unit
NOK: Norwegian kroner
OAEOC: Oslo Accident and Emergency Outpatient Clinic
USD: US dollar
