# Supplementary material for: Treating patients with opioid overdose at a primary care emergency outpatient clinic: a cost-minimization analysis
Source: Cost Eff Resour Alloc. 2021 Aug 4;19:48. doi: 10.1186/s12962-021-00303-6 (PMC8335998; doi:10.1186/s12962-021-00303-6)
Supplement: Supplementary file 1 — Additional file 1: Table S1. Blood tests at hospital. [file 12962_2021_303_MOESM1_ESM.docx]

**Table S1. Blood tests at hospital**

B-Erythrocyte sedimentation rate

B-Haematocrit

B-Erythrocytes

B-Mean cell volume

B-Mean cell haemoglobin

B-Leukocytes

B-Neutrophils

B-Lymphocytes

B-Monocytes

B-Eosinophils

B-Platelets

S-Sodium

S-Potassium

S-Calcium

S-Creatinine

S-Cystatin C

S-Alanine aminotransferase

S-Alkaline phosphatase

S-Bilirubin

S-Glucose

S-Albumin
