# Supplementary material for: Treating patients with opioid overdose at a primary care emergency outpatient clinic: a cost-minimization analysis
Source: Cost Eff Resour Alloc. 2021 Aug 4;19:48. doi: 10.1186/s12962-021-00303-6 (PMC8335998; doi:10.1186/s12962-021-00303-6)
Supplement: Supplementary file 2 — Additional file 2: Figure S1. Decision tree at outpatient clinic. [file 12962_2021_303_MOESM2_ESM.pdf]

Supplementary figure 1. Decision tree at outpatient clinic

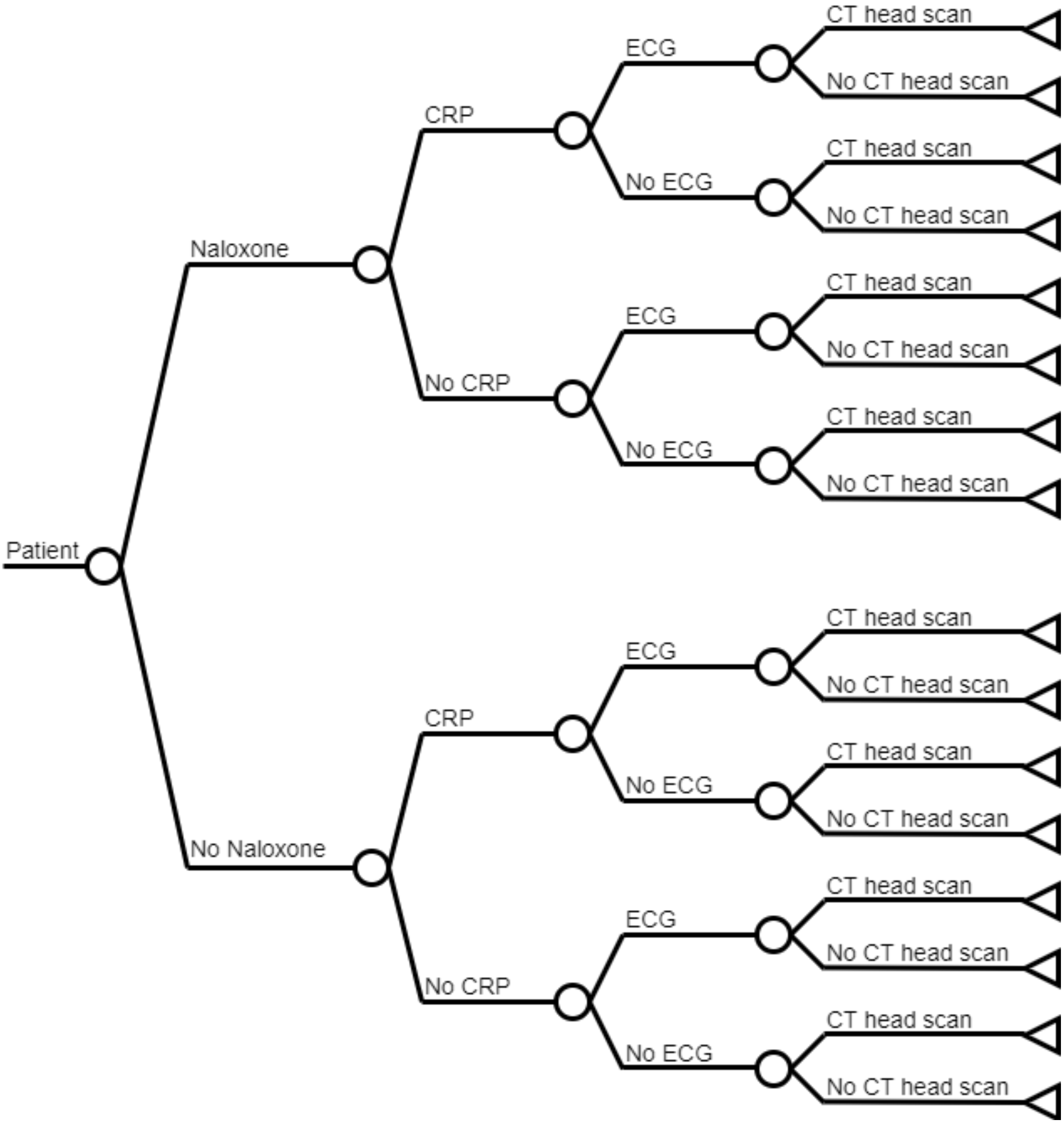

The graph shows combinations of tests and treatments at the OAEOC. The probabilities from Table 1 were used at all relevant chance nodes (e.g., at baseline, the probability of a CT head scan was 6 % at each of the eight chance nodes in the figure).
Glucose (Table 1) was excluded from the graph but included in the calculations.
CRP: C-reactive protein; CT: computed tomography; ECG: electrocardiogram; OAEOC: Oslo Accident and Emergency Outpatient Clinic.
