# Supplementary material for: Treating patients with opioid overdose at a primary care emergency outpatient clinic: a cost-minimization analysis
Source: Cost Eff Resour Alloc. 2021 Aug 4;19:48. doi: 10.1186/s12962-021-00303-6 (PMC8335998; doi:10.1186/s12962-021-00303-6)
Supplement: Supplementary file 3 — Additional file 3: Figure S2. Decision tree at hospital. [file 12962_2021_303_MOESM3_ESM.pdf]

Supplementary figure 2. Decision tree at hospital

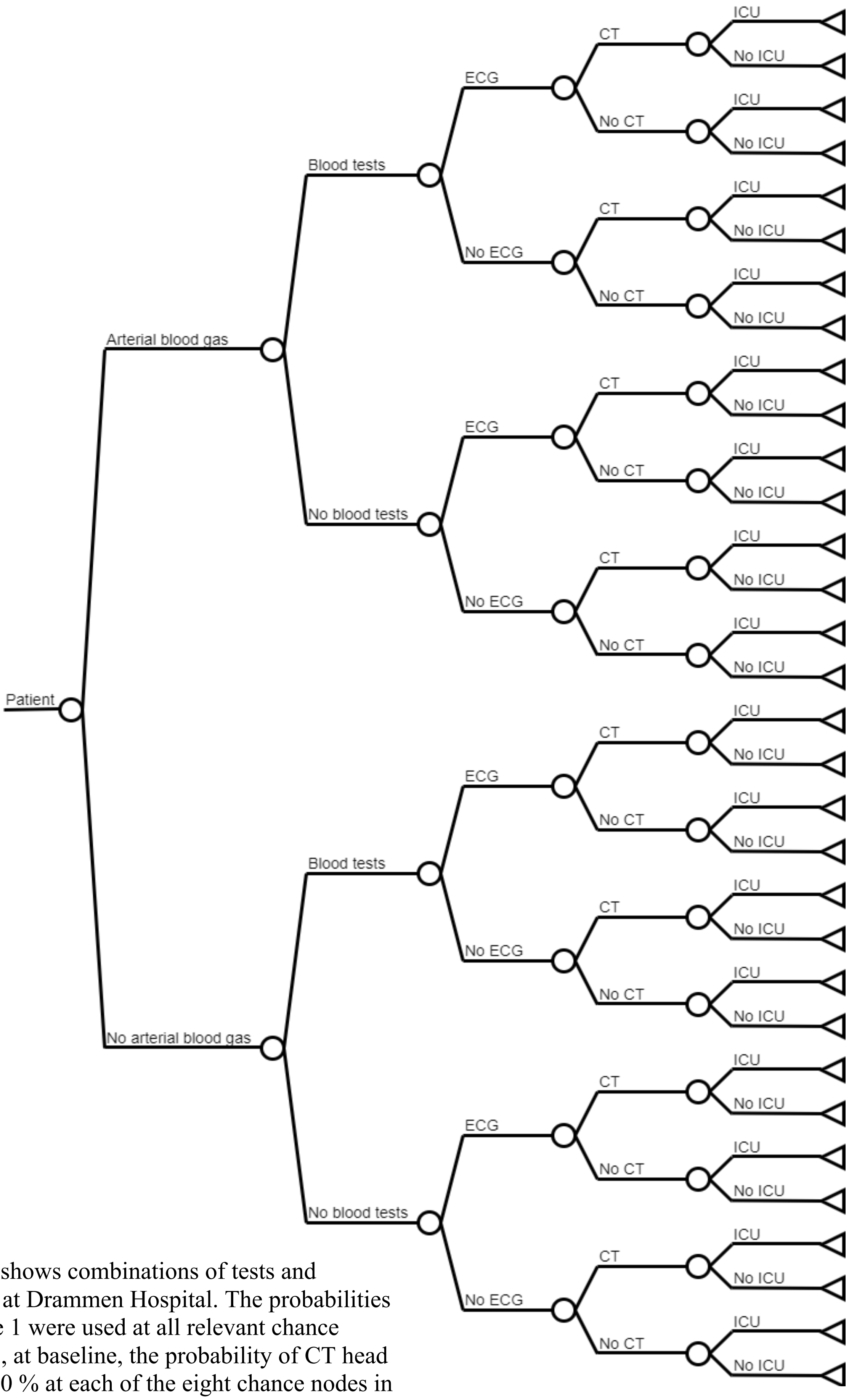

The graph shows combinations of tests and treatments at Drammen Hospital. The probabilities from Table 1 were used at all relevant chance nodes (e.g., at baseline, the probability of CT head scan was 40 % at each of the eight chance nodes in the figure).
Naloxone, flumazenil, and Ringer acetate (Table 1) were excluded from the graph but included in the calculations.

CT: computed tomography head scan; ECG: electrocardiogram; ICU: intensive care unit.
